# Convolutional neural network-based vocal cord tumor classification technique for home-based self-prescreening purpose

**DOI:** 10.1186/s12938-023-01139-2

**Published:** 2023-08-18

**Authors:** Gun Ho Kim, Young Jun Hwang, Hongje Lee, Eui-Suk Sung, Kyoung Won Nam

**Affiliations:** 1 Medical Research Institute, Pusan National University, Yangsan, Korea; 2 Department of Biomedical Engineering, Pusan National University Yangsan Hospital, Yangsan, Korea; 3 Department of Biomedical Engineering, School of Medicine, Pusan National University, 49, Busandaehak-Ro, Mulgeum-Eup, Yangsan, 50629 Korea; 4 Department of Nuclear Medicine, Dongnam Institute of Radiological & Medical Sciences, Busan, Korea; 5 Department of Otolaryngology-Head and Neck Surgery, Pusan National University Yangsan Hospital, Yangsan, Korea; 6 Department of Otolaryngology-Head and Neck Surgery, School of Medicine, Pusan National University, Yangsan, Korea; 7 Research Institute for Convergence of Biomedical Science and Technology, Pusan National University Yangsan Hospital, Yangsan, Korea

**Keywords:** Vocal cord tumor, Convolutional neural network, Otolaryngology, Deep learning

## Abstract

**Background:**

In this study, we proposed a deep learning technique that can simultaneously detect suspicious positions of benign vocal cord tumors in laparoscopic images and classify the types of tumors into cysts, granulomas, leukoplakia, nodules and polyps. This technique is useful for simplified home-based self-prescreening purposes to detect the generation of tumors around the vocal cord early in the benign stage.

**Results:**

We implemented four convolutional neural network (CNN) models (two Mask R-CNNs, Yolo V4, and a single-shot detector) that were trained, validated and tested using 2183 laryngoscopic images. The experimental results demonstrated that among the four applied models, Yolo V4 showed the highest F1-score for all tumor types (0.7664, cyst; 0.9875, granuloma; 0.8214, leukoplakia; 0.8119, nodule; and 0.8271, polyp). The model with the lowest false-negative rate was different for each tumor type (Yolo V4 for cysts/granulomas and Mask R-CNN for leukoplakia/nodules/polyps). In addition, the embedded-operated Yolo V4 model showed an approximately equivalent F1-score (0.8529) to that of the computer-operated Yolo-4 model (0.8683).

**Conclusions:**

Based on these results, we conclude that the proposed deep-learning-based home screening techniques have the potential to aid in the early detection of tumors around the vocal cord and can improve the long-term survival of patients with vocal cord tumors.

## Background

Vocal cords are the folds of tissues related to voice creation. Benign tumors, such as cysts, granulomas, leukoplakia, nodules, and polyps, generated in the vicinity of the vocal cord can induce several clinical complications, including wheezing, stridor, dysphonia, cough, asthma, and vocal cord palsy, and progress to malignant tumors or even cancer. Similar to other tumors generated in the oral, laryngeal, and oropharyngeal positions, early detection and timely treatment of vocal cord tumors can improve the 5-year survival rate of patients. However, unlike tumors around the tongue or gums, early stage tumors around the vocal cord are not easily detected with the naked eye, because many patients with vocal cord tumors first visit a hospital after the tumors have already progressed to malignancy or even cancer. In addition, it can be more helpful for individuals if information about the specific type of vocal cord tumors is provided during the first detection of the tumor in its benign stage, because the plan for medical treatment can differ according to the type of vocal cord tumors; for example, direct surgical treatment is recommended for cysts, leukoplakia, and polyps; however, voice therapy is first recommended in cases of granuloma and nodules [1]. In particular, most initial-stage nodules improve with voice therapy; by contrast, approximately 50% of leukoplakia cases are dysplastic and require early surgical treatment [2]. Therefore, to improve the long-term survival of patients with vocal cord tumors, it is necessary to develop a home-based vocal cord self-screening technique that can easily detect the generation of early stage tumors around the vocal cord and also provide the specific type of vocal cord tumors detected during the self-screening procedure as an initial guide for proper treatments.

In this study, we used four popular convolutional neural network (CNN) models for endoscopic vocal cord images to detect the positions of tumor-suspicious areas in the image and simultaneously provide the specific types of vocal cord tumors within five subclasses (cyst, granuloma, leukoplakia, nodule, and polyp). We further ported a CNN model that exhibited the best performance among the four applied models in a high-performance computing environment to operate in an embedded environment and verified the clinical usability of the model as a tool for home-based self-prescreening.

## Related works

Recently, owing to the rapid development of artificial intelligence (AI) techniques, many studies have applied deep learning (DL) techniques to diagnose various medical images, such as X-rays, computed tomography, and magnetic resonance devices, because these imaging devices can provide standardized and high-resolution images, which are convenient for most AI-diagnostic studies [3]. By contrast, the number of DL studies based on endoscopic images is relatively small, and most target gastrointestinal and colorectal endoscopic images [4–6]. Most studies have analyzed oral, laryngeal, and oropharyngeal endoscopic images using traditional image-processing techniques [7, 8]. However, several machine learning- or deep-learning-based disease classification studies targeting the vocal areas have been reported [9, 10]. For example, Ren et al. applied ResNet-101 to classify laryngoscopic vocal images, including normal, nodule, polyp, leukoplakia and malignancy [11]; Zhao et al. applied MobileNet-V2 to classify vocal cord lesions (normal, polyp, keratinization and carcinoma) [12]; Byeon compared the performance of five machine learning and deep learning models (deep learning, naive Bayes model, generalized linear model, classification/regression tree and random forest) for predicting benign laryngeal mucosal disorders (nodules, polyps, cysts, Reinke’s edema, granuloma, glottic sulcus and laryngeal keratosis) [13]; Larsen et al*.* applied four CNN models (five-layer CNN, VGG19, MobileNet-V2, and InceptionResNet-V2) to classify the images into abnormal (nodules) and normal [14]. In addition, Cho et al. reported the following two related papers: in one study, they applied four CNN models (six-layer CNN, VGG16, Inception-V3 and Xception) to laryngoscopic vocal fold images to classify the image into abnormal and normal [15], and in the other study, they applied four CNN models (VGG16, Inception-V3, MobileNet-V2 and EfficientNet-B0) to classify laryngeal diseases (cysts, nodules, polyps, leukoplakia, papillomas, Reinke’s edema, granulomas, palsies and normal) [16]; You et al. applied 13 CNN models (AlexNet, four VGG models, three ResNet models, three DenseNet models, Inception-V3, and the proposed) to classify laryngeal leukoplakia (inflammatory keratosis, mild/moderate/severe dysplasia, and squamous cell carcinoma) using white-light endoscopy images [17]; Eggert et al. applied DenseNet models to classify hyperspectral images of laryngeal, hypopharyngeal, and oropharyngeal mucosa into abnormal and normal [18]. Moreover, Hu et al. applied Mask R-CNN with ResNet-50 backbone to two types of laryngoscopic imaging (narrow-band imaging and white-light imaging) for automated real-time segmentation and classification of vocal cord leukoplakia to classify the lesions into surgical and non-surgical groups [19]; Yan et al. applied the Faster R-CNN model to laryngoscopic images of vocal lesions to screen for laryngeal carcinoma [20]; Kim et al. applied the Mask R-CNN model to laryngoscopic images for real-time segmentation of laryngeal mass around the vocal cord [21]; Cen et al. applied three CNN models (Faster R-CNN, Yolo V3, and SSD) to detect laryngeal tumors in endoscopic images (vocal fold, tumor, surgical tools, and other laryngeal tissues) [22]; Azam et al. applied up to nine Yolo models to laryngoscopic video for real-time detection of laryngeal squamous cell carcinoma in both white-light and narrow-band imaging [23]. Among these previous studies on vocal area disease detection, eight [11–18] used AI models for classification and, therefore, were not able to provide information about the tumor-suspicious positions in the image. Similar to the current study, five other studies [19–23] used AI models for object detection that can provide tumor-suspicious positions around the vocal cords; however, they commonly used only single-group disease images, such as vocal cord leukoplakia [19], laryngeal carcinoma [20, 23], laryngeal mass [21], and cancer [22].

In addition, several studies have reported the use of personal IT devices to detect oral diseases in their early stages. For example, Askarian et al. proposed a k-nearest neighborhood-based strep-throat classification algorithm using smartphone camera images [24]. Song et al. proposed a CNN-based oral cancer detection algorithm using a smartphone-based intraoral dual-modality imaging platform [25]. Yoo et al. proposed three CNN models (ResNet-50, Inception-V3 and MobileNet-V2) that can detect severe pharyngitis using throat images captured using a smartphone [26]. The target area of these studies was the back of the throat, including the tonsils [24], palate of a healthy patient, buccal mucosa of a patient with potentially oral malignant lesions, and malignant lesions from the lower vestibule [25] and throat [26]; however, none of these studies targeted tumors around the vocal cords. In addition, all these studies adopted a binary classification structure that can classify the input image only as healthy (normal) or diseased (suspicious), with none showing the positions of tumor-suspicious areas around the vocal cord in the image and, at the same time, providing more detailed classification results for the suspicious areas.

Therefore, to improve the clinical usability of the home-based vocal cord self-screening technique, the self-screening application should provide more information about the positions of the suspicious areas and the specific type of each suspicious area, as well as the results of the binary classification.

## Result

**Fig. 1:**
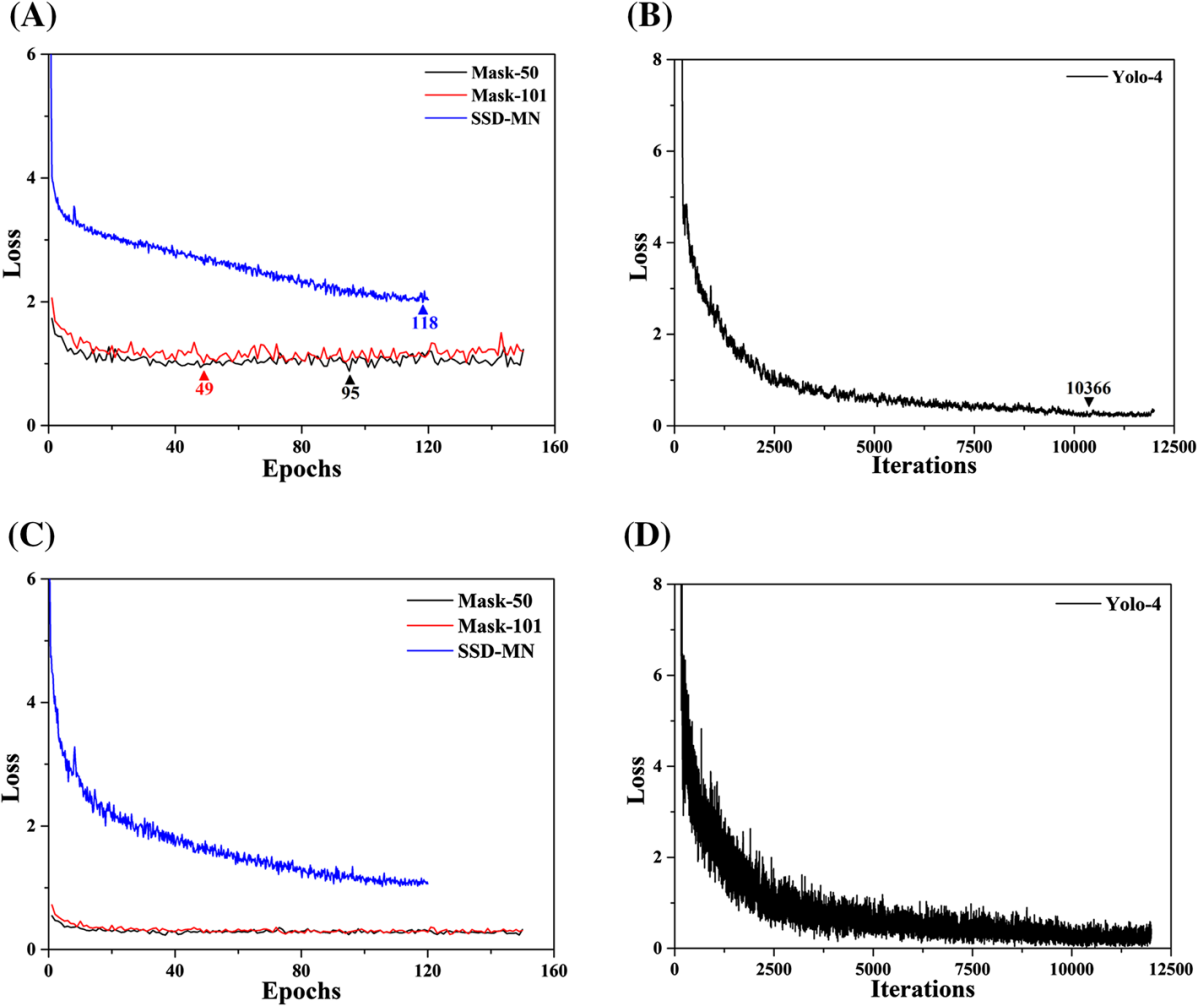
Curves of validation and bounding box loss in accordance with the increase of the epoch/iteration values (using validation data set). **A** Validation loss of Mask-50 (black), Mask-101 (red) and SSD-MN (blue). **B** Validation loss of Yolo-4. **C** Bounding box loss of Mask-50 (black), Mask-101 (red) and SSD-MN (blue). **D** Bounding box loss of Yolo-4

Figure 1A, B presents the validation loss curves for the Mask-50/Mask-101 (epoch range: 1–150) [27], Yolo-4 (iteration range: 1–12,000) [28], and SSD-MN (epoch rage: 1–120) [29] models. Figure 1C, D shows the bounding box loss curves for the same models. Based on these results, we selected epoch/iteration values of 95, 49, 10,366, and 118 for the Mask-50, Mask-101, Yolo-4, and SSD-MN models, respectively, during confusion matrix analysis using the test data set.

**Fig. 2:**
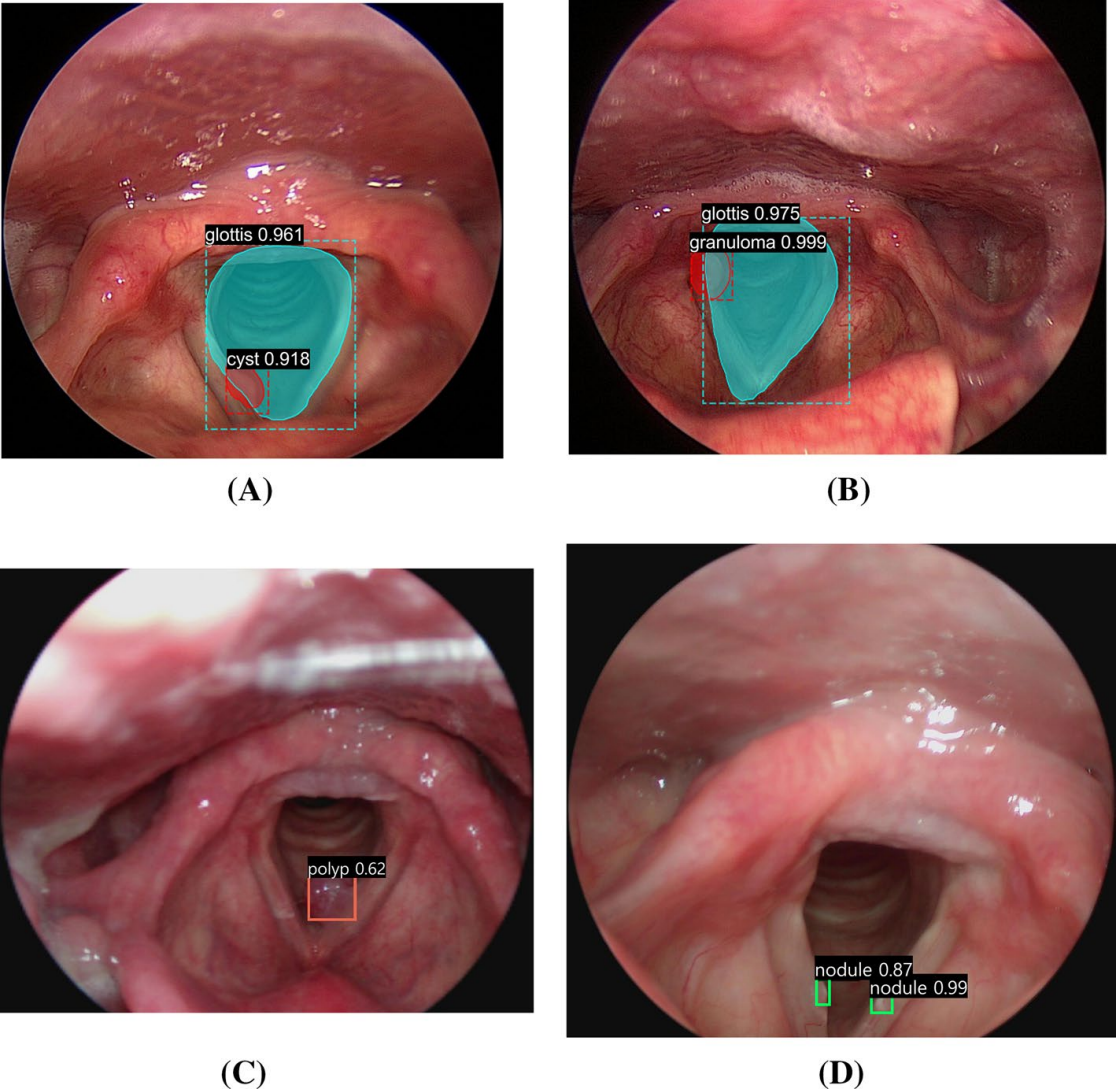
Results of simultaneous tumor-detection and type-classification using the four implemented CNN models (for sample images in test data set). **A** Mask-50—cyst. **B** Mask-101—granuloma. **C** SSD-MN—polyp. **D** Yolo-4—nodule

Figure 2 shows the results of the simultaneous tumor detection and type-classification using the four implemented CNN models and sample images of cysts, granulomas, polyps, and nodules in the test data set. The Mask-50/Mask-101 models display both bounding box areas and segmentation areas, whereas the SSD-MN and Yolo-4 models display bounding box areas, which were all matched with the results of annotation by a clinical expert.

**Fig. 3:**
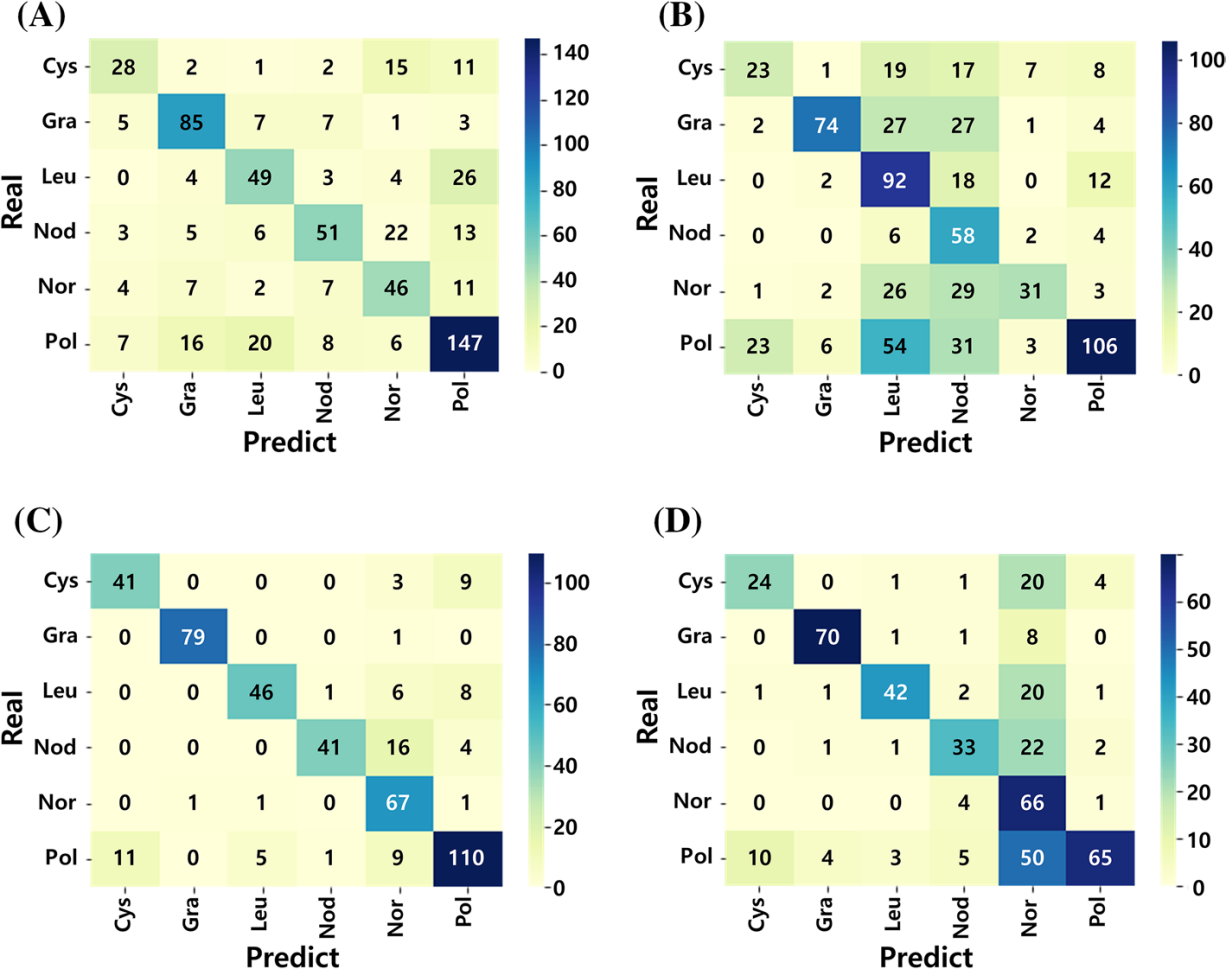
Results of the class determination for test data set images. **A** Mask-50. **B** Mask-101. **C** Yolo-4. **D** SSD-MN. *Cys* Cyst, *Gra* Granuloma, *Leu* Leukoplakia, *Nod* Nodule, *Nor* Normal, *Pol* Polyp

Figure 3 shows the class determination results of each CNN model for the test data set images, and Table 1 lists the results of the confusion matrix analysis of the four CNN models for each tumor type. For all types of benign tumors, the values of the F1-score, which represent the overall performance of the model, were the highest for the Yolo-4 model among the four applied models: 0.7664 for cysts, 0.9875 for granulomas, 0.8214 for leukoplakia, 0.8119 for nodules, and 0.8271 for polyps. In the case of false-negative (FN), which can further represent the feasibility of the model as a tool for home-based pre-screening purposes, the models with the lowest FN values were as follows: Yolo-4, cyst (14) and granuloma (1); Mask-101, leukoplakia (7) and nodule (14); and Mask-50, polyp (18).

**Table 1:** Results of the confusion matrix analysis of the four convolutional neural network models for each tumor type

Tumor	Model	TP	FP	FN	TN	Acc	Pre	Rec	Spe	F1
Cyst	Mask-50	28	19	25	299	0.8814	0.5957	0.5283	0.9403	0.5600
	Mask-101	25	24	30	291	0.8541	0.5102	0.4545	0.9238	0.4808
	Yolo-4	41	11	**14**	302	0.9321	0.7885	0.7455	0.9649	**0.7664**
	SSD-MN	24	11	30	301	0.8880	0.6857	0.4444	0.9647	0.5393
Granuloma	Mask-50	85	34	2	262	0.9060	0.7143	0.9770	0.8851	0.8252
	Mask-101	74	11	8	278	0.9488	0.8706	0.9024	0.9619	0.8862
	Yolo-4	79	1	**1**	287	0.9946	0.9875	0.9875	0.9965	**0.9875**
	SSD-MN	70	6	8	281	0.9616	0.9211	0.8974	0.9791	0.9091
Leukoplakia	Mask-50	49	36	21	277	0.8512	0.5765	0.7000	0.8850	0.6323
	Mask-101	92	132	**7**	226	0.6958	0.4107	0.9293	0.6316	0.5697
	Yolo-4	46	6	14	303	0.9458	0.8846	0.7667	0.9806	**0.8214**
	SSD-MN	42	6	21	302	0.9272	0.8750	0.6667	0.9805	0.7568
Nodule	Mask-50	51	27	14	296	0.8943	0.6538	0.7846	0.9164	0.7133
	Mask-101	61	119	**14**	246	0.6977	0.3387	0.8133	0.6740	0.4784
	Yolo-4	41	2	17	313	0.9491	0.9535	0.7069	0.9937	**0.8119**
	SSD-MN	33	13	24	306	0.9016	0.7174	0.5789	0.9592	0.6408
Polyp	Mask-50	146	65	**18**	190	0.8019	0.6919	0.8902	0.7451	0.7787
	Mask-101	105	32	36	210	0.8225	0.7664	0.7447	0.8678	0.7554
	Yolo-4	110	22	24	218	0.8770	0.8333	0.8209	0.9083	**0.8271**
	SSD-MN	64	9	68	229	0.7919	0.8767	0.4848	0.9622	0.6244

Bold values in the table represent the cases of the lowest error (lowest in false negative) and the best performance (highest in F1-score) among the four models

*TP* true-positive, *FP* false-positive, *FN* false-negative, *TN* true-negative, *Acc* accuracy, *Pre* precision, *Rec* recall, *Spe* specificity, *F1* F1-score

Table 2 shows the results of the confusion matrix analysis of the four CNN models for all healthy and benign cases. For all test data sets, Yolo-4 showed the highest F1-score (0.8499), accuracy (0.9395), precision (0.8830), recall (0.8191), specificity (0.9713), and lowest FN value (70) among the four applied models.

**Table 2:** Results of the confusion matrix analysis of the four convolutional neural network models for overall healthy and benign cases

	TP	FP	FN	TN	Acc	Pre	Rec	Spe	F1
Mask-50	359	181	80	1324	0.8657	0.6648	0.8178	0.8797	0.7334
Mask-101	357	318	95	1251	0.7956	0.5289	0.7898	0.7973	0.6335
Yolo-4	317	42	**70**	1423	0.9395	0.8830	0.8191	0.9713	**0.8499**
SSD-MN	233	45	151	1419	0.8939	0.8381	0.6068	0.9693	0.7039

Bold values in the table represent the cases of the lowest error (lowest in false negative) and the best performance (highest in F1-score) among the four models

*TP* true-positive, *FP* false-positive, *FN* false-negative, *TN* true-negative, Acc accuracy, *Pre* precision, *Rec* recall, *Spe* specificity *F1* F1-score

**Fig. 4:**
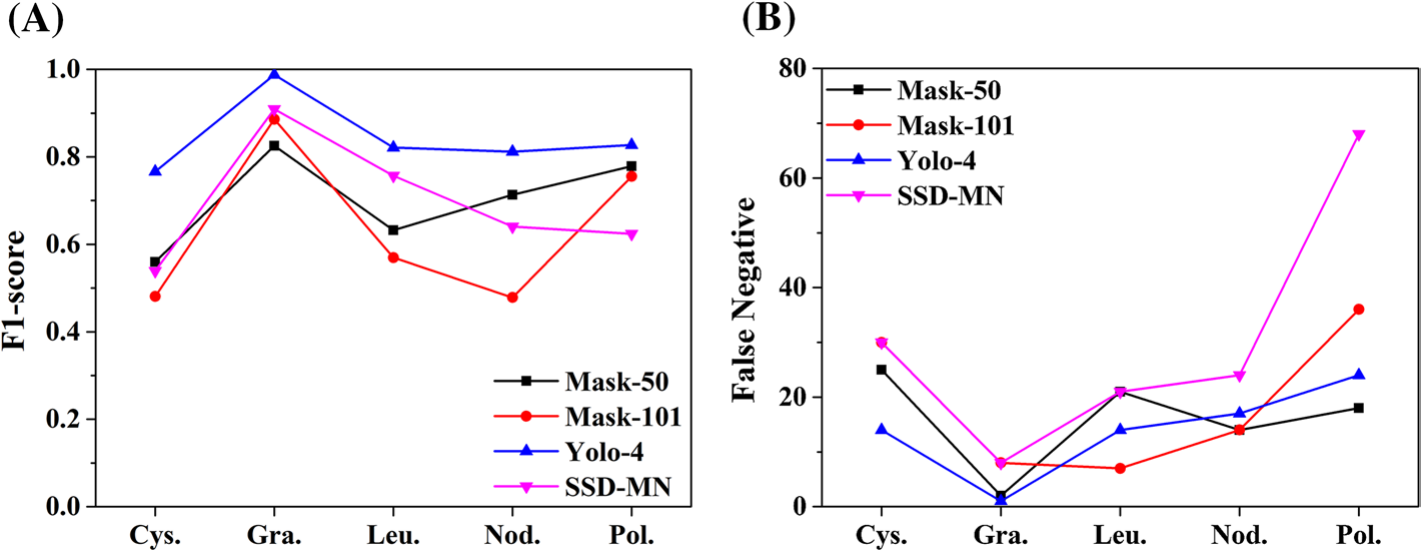
Ranks of each CNN models (Mask-50, Mask-101, Yolo-4, and SSD-MN) for each type of vocal cord tumor in aspects of F1-score (**A**) and false-negative (**B**). *Cys* Cyst, *Gra* Granuloma, *Leu* Leukoplakia, *Nod* Nodule, *Pol* Polyp

Figure 4 shows the ranks of each CNN model for each type of vocal cord tumor in terms of the F1-score and FN results. Considering these graphs and the experimental results shown in Table 2, we concluded that for our data set, the Yolo-4 model is the most suitable CNN model for home-based prescreening for the early detection of benign vocal cord tumors.

Table 3 presents a comparison between the computer-operated Yolo-4 model with the original images and the embedded-operated Yolo-4 model with the camera images for 100 randomly selected test data set images. The embedded-operated Yolo-4 model showed an approximately equivalent classification performance to that of the computer-operated Yolo-4 model, which implied the clinical feasibility of the implemented AI model as a tool for home-based self-prescreening.

**Table 3:** Results of the comparison test between the computer-operated Yolo-4 model and the embedded-operated Yolo-4 model for 100 randomly selected test data set images

Platform	TP	FP	FN	TN	Acc	Pre	Rec	Spe	F1
Computer	89	9	18	391	0.9467	0.9082	0.8318	0.9775	0.8683
Embedded	87	9	21	391	0.9409	0.9063	0.8056	0.9775	0.8529

*TP* true-positive, *FP* false-positive, *FN* false-negative, *TN* true-negative, *Acc* accuracy, *Pre* precision, *Rec* recall, *Spe* specificity, *F1* F1-score

## Discussion

The four CNN models implemented in the current study can provide information about tumor-suspicious positions around the vocal cord and, simultaneously, provide more detailed classification results (cysts, granulomas, leukoplakia, nodules, and polyps), which is an advantage of the current study compared with previous studies. In addition, we evaluated the clinical feasibility of the implemented CNN model as a tool for home-based self-prescreening by porting a computer-based model onto a popular embedded device. Experimental results from the embedded device demonstrated the potential of the implemented model to assist in the early detection of tumors generated in the vicinity of the vocal cords by individuals at home.

In this study, we aimed to develop a reliable diagnostic support technique using deep learning that can be easily utilized outside hospitals by non-medical experts for self-prescreening purposes. This kind of at-home self-prescreening technique can be particularly helpful and promising when highly infectious diseases such as COVID-19 are spreading, because many people are unwilling to visit hospitals for checkups. As a result, early detection of vocal cord tumors has become more difficult. Currently, a popular embedded device is used to implement an at-home self-prescreening platform. If the implemented AI model is further ported to operate on a smartphone, the transmission of smartphone-photographed vocal cord images to a remote hospital server or cloud can be enabled. In addition to at-home self-prescreening based on the AI technique, more detailed diagnostic results related to the image, such as periodic tumor progression monitoring, counseling, and prescription, can be provided by a medical expert to an individual via a smartphone, without frequent visits to the hospital. To implement such a system, it is necessary to gather more endoscopic images of benign, malignant, and cancerous lesions around the vocal cord and improve the currently implemented AI models, which is one of our future research topics.

This study has some limitations. First, we used 2183 images from the hospital database, which was insufficient for training the deep learning model, because the more images used during the training and validation phases, the better the quality of at-home self-prescreening of oral/laryngeal tumors. To further improve the performance and reliability of the AI model, it is necessary to conduct additional multi-country multi-city (MCC) collaborative research with various hospitals to gather more diagnostic images of various oral and laryngeal areas in future studies. Second, we downloaded the sample codes for the three CNN models (Mask R-CNN, Yolo, and SSD) from GitHub and partially modified them to fit our study purposes. To further enhance the model performance and reduce its hardware requirements, which are necessary for reliable real-time on-device AI operations, it is necessary to optimize the current model codes in further studies. In addition, although the implemented models showed reasonable performance on a utilized embedded platform, there are an increasing number of lightweight state-of-the-art models, such as EfficientDet/EfficientNet, BASIC-L, and InternImage-H, which are suitable for at-home self-prescreening of the oral and laryngeal regions. To improve the net value of the present study, it is necessary to further implement and apply more recent models to find the most suitable deep learning model for at-home self-prescreening of the oral and laryngeal regions and to further improve the performance and operation time of the selected models on a lighter embedded device in future studies. Third, we evaluated the performance of the implemented CNN models in terms of confusion matrix analysis, because the primary target of the current study was to show the performance equivalence between computer and embedded environments (Table 3); however, to improve the clinical feasibility of the current study (i.e., optimize the current models or replace other higher performance models), it is also necessary to further verify the performance of the implemented CNN models in terms of intersection over union, mean average precision, area under the region-of-interest curve, precision–recall graph, and mean inference time in future studies.

## Conclusion

In this study, we evaluated the possibility of a deep-learning-based endoscopic image analysis technique for at-home self-prescreening of vocal cord tumors by non-medical experts. Based on the experimental results, we concluded that the implemented deep learning models have the potential to aid in the early detection of tumors near the vocal cord, which may improve the long-term survival of patients with vocal cord tumors.

## Materials and methods

### Data preparation

**Fig. 5:**
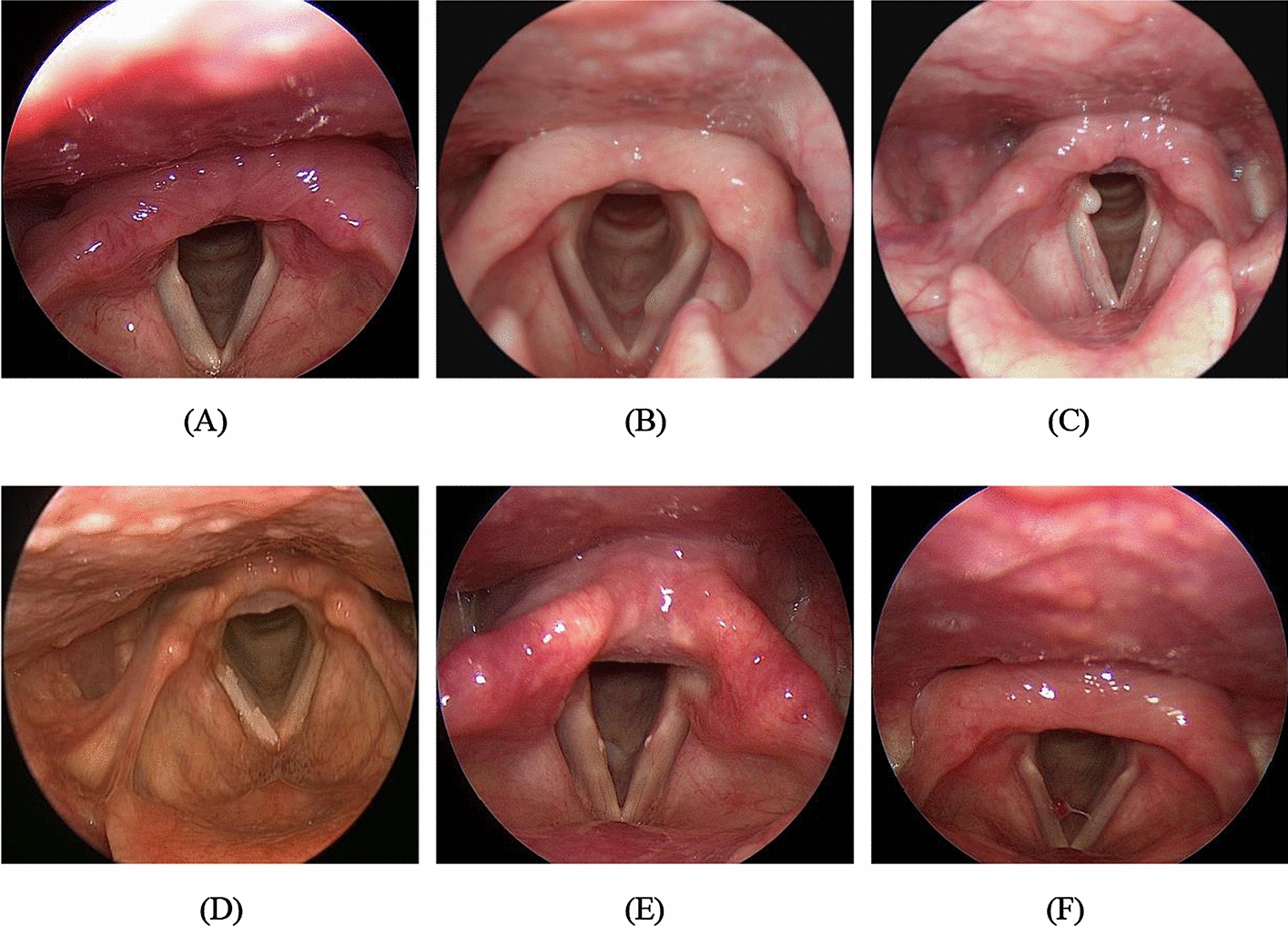
Examples of the endoscopic vocal cord images (test data set) for healthy group (normal) and benign group (cyst, granuloma, leukoplakia, nodule, and polyp). **A** Normal. **B** Cyst. **C** Granuloma. **D** Leukoplakia. **E** Nodule. **F** Polyp

We acquired 2183 laryngoscopic images (349 from the healthy group and 1834 from the benign group) from the Picture Archiving and Communication System of Pusan National University Yangsan Hospital after IRB approval (No. 05-2019-008). A trained otolaryngologist acquired, classified, and labeled the imaging data. All acquired images were unidentified before the model application. The images in the benign group were further divided into the following five subgroups: cysts, 242 images; granulomas, 386 images; leukoplakia, 291 images; nodules, 256 images; and polyps, 657 images. The acquired images were then divided into training, validation, and test data sets at a 3:1:1 ratio (Fig. 5).

### Model implementation

**Fig. 6:**
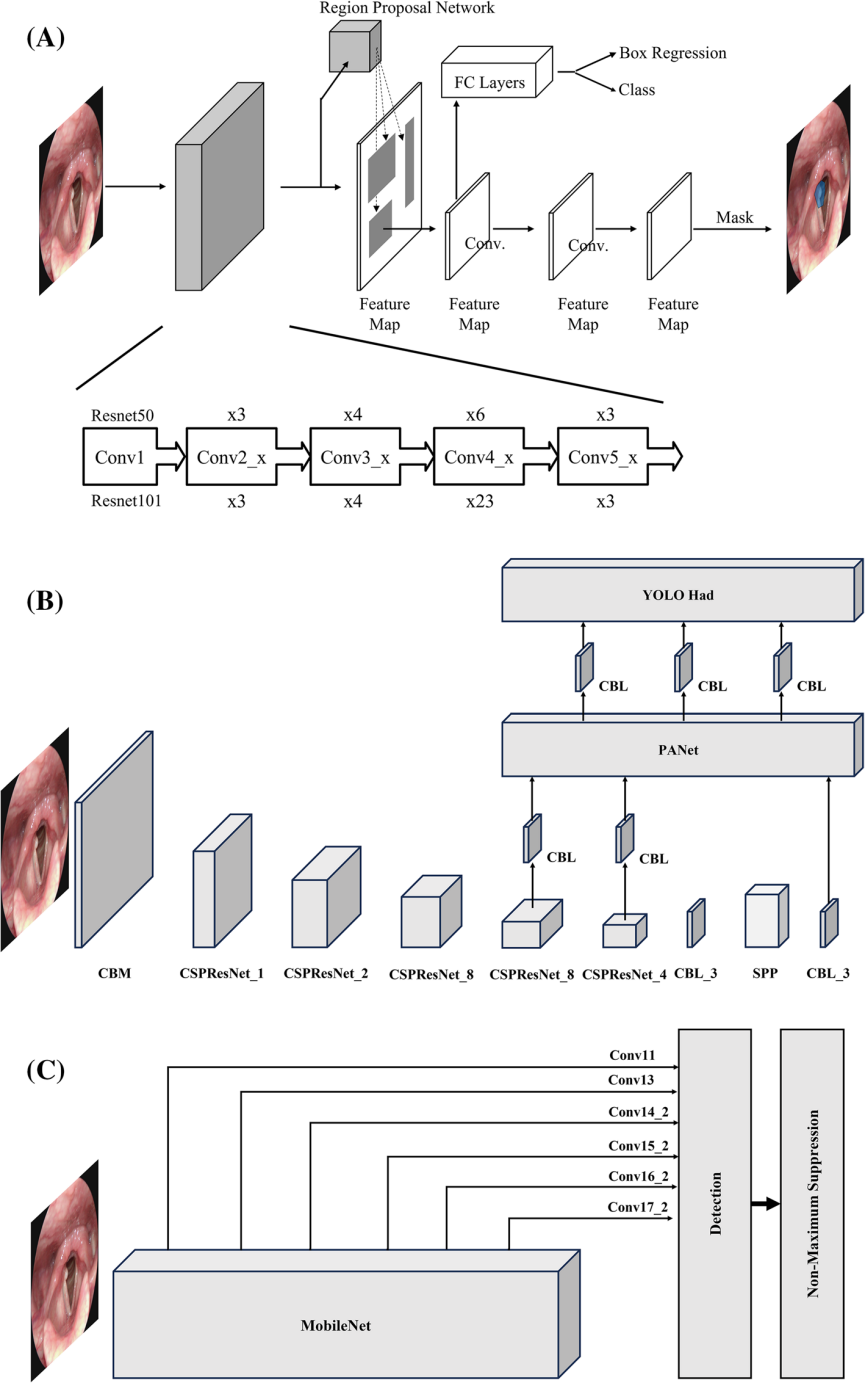
Flow diagrams of the implemented CNN models. **A** Mask-50 and Mask-101. **B** Yolo-4 **C** SSD-MN

We implemented four CNN models that can detect the position of tumor-suspicious areas and classify the type of tumors in the suspicious areas: Mask R-CNN with ResNet-50 backbone (Mask-50), Mask R-CNN with ResNet-101 backbone (Mask-101), Yolo V4 (Yolo-4) and a single-shot detector with a MobileNet backbone (SSD-MN). Figure 6 presents the flow diagrams of the implemented CNN models. For this study, we downloaded sample codes of four CNN models from GitHub and modified them to fit our research and system environments [27–29]. Table 4 provides detailed information regarding the model development environments.

**Table 4:**
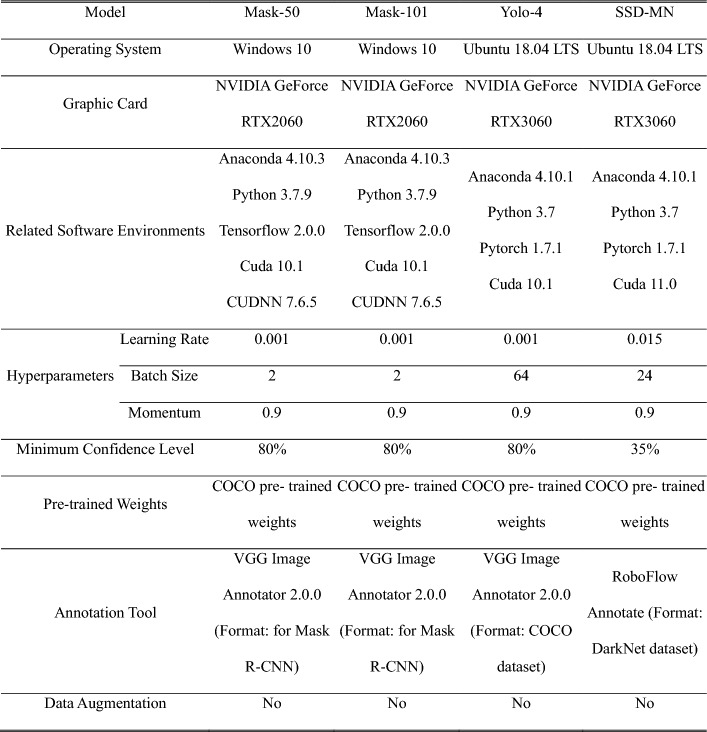
Detailed information about the model development environments

### Model evaluation

We performed confusion matrix analysis using 374 test images to quantitatively evaluate the performance of the applied CNN models. The definitions of the true-positive (TP), false-positive (FP), true-negative (TN), and false-negative (FN) were as follows: (1) TP: both the model-determined position and type of the tumor coincided with those of the expert labeling; (2) FP: one or both of the model-determined position and type of the tumor did not coincide with that of the expert labeling; (3) TN: the model-determined images from the healthy group were normal; and (4) FN: the model-determined images from the benign group were normal. The accuracy, precision, recall, specificity, and F1-score (harmonic mean between precision and recall) were calculated as follows:

$$Accuracy \,\left(Acc\right)= \frac{TP+TN}{TP+FP+FN+TN}$$

$$Precision \,\left(Pre\right)= \frac{TP}{TP+FP}$$

$$Recall \,\left(Rec\right)= \frac{TP}{TP+FN}$$

$$Specificity \,\left(Spe\right)= \frac{TN}{FP+TN}$$

1 $$F1\text-Score=\frac{2\times Pre\times Rec}{Pre+Rec}$$

**Fig. 7:**
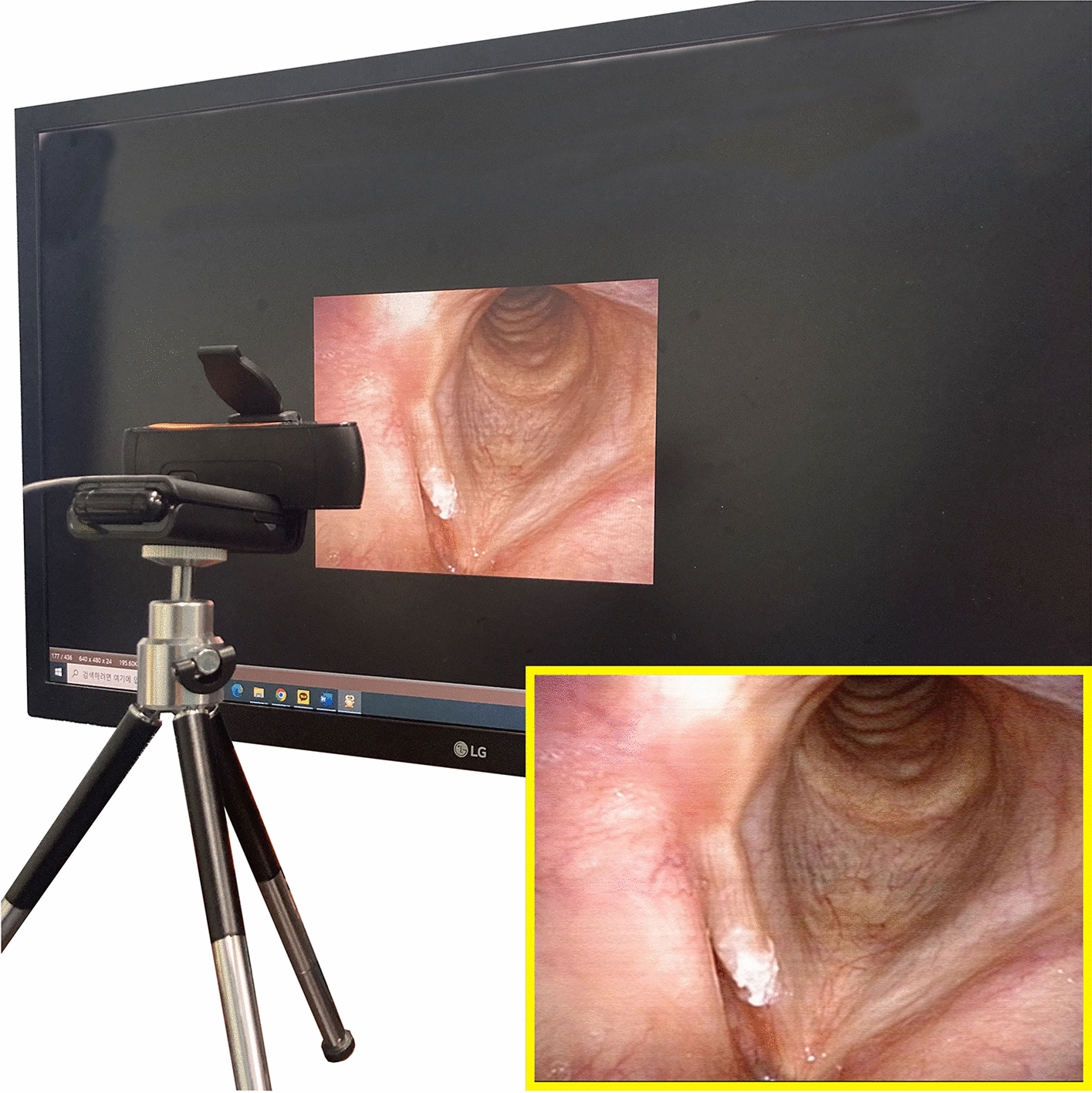
Evaluation of the embedded-ported convolutional neural network model using a web camera. The image in the yellow rectangular contour represents the web camera-photographed image

In addition, to further verify the feasibility of the implemented CNN model as a tool for home-based self-prescreening to detect early benign tumors around the vocal cord, we ported a CNN model that showed the best performance during confusion matrix analysis of the computer environment to operate on a popular embedded system (NVDIA Jetson Nano^™^ Developer Kit; NVIDIA Tegra X1, Python 3.6, CUDA 10.2, CUDNN 8.2.1, Opencv 4.1.1, and JetPack 4.6.1). A web camera (C922 Pro Stream^™^; Logitech International S.A., Lausanne, Switzerland; 1920 × 1080,) was connected via a USB port, a 32-in monitor (UltraGear 32GK650F; LG Electronics Inc., Seoul, Korea) was connected via an HDMI port, and the web camera was positioned in front of the monitor (Fig. 7). One hundred images in the test data set were randomly selected and displayed on the screen individually (monitor setting: QHD 2560 × 1440 resolution, 144 Hz refresh rate, 350 cd/m^2^ brightness, NTSC 72% color gamut, 70% in 3000:1 contrast ratio), and the web camera captured the images on the screen (camera setting: 1920 × 1080 resolution, FHD 1080p/30fps, 78° field of view; focus and brightness were automatically adjusted).

## Data Availability

Not applicable.

## Abbreviations

CNN: Convolutional neural network
AI: Artificial intelligence
Mask-50: Mask R-CNN with ResNet-50 backbone
Mask-101: Mask R-CNN with ResNet-101 backbone
Yolo-4: Yolo V4
SSD-MN: Single-shot detector with MobileNet backbone
TP: True-positive
TN: True-negative
FP: False-positive
FN: False-negative
